# Editorial: Antidiabetic molecular targets: Updates on old and emerging targets and their small molecule modulators

**DOI:** 10.3389/fchem.2023.1150026

**Published:** 2023-02-06

**Authors:** Md. Shahidul Islam, Parvesh Singh, Kamaldeep Paul

**Affiliations:** ^1^ Department of Biochemistry, School of Life Sciences, University of KwaZulu-Natal, Durban, South Africa; ^2^ Department of Chemistry, School of Chemistry and Physics, University of KwaZulu-Natal, Durban, South Africa; ^3^ School of Chemistry and Biochemistry, Thaper Institute of Engineering and Technology, Patiala, India

**Keywords:** type 2 diabetes, prediabetes, gestational diabetes, small molecules, micro RNA, D-chiro-inositol

Considering the rapidly growing prevalence of diabetes around the world (IDF, 2021) and gradually changing pathogenesis of type 2 diabetes (T2D) (Kautzky-Willer et al., 2016), there has been an increasing interest in the discovery and synthesis of novel antidiabetic molecules as well as to understand their molecular targets and underlying molecular mechanism of actions. Recently a number of compounds both from natural and synthetic origins have been investigated for their antidiabetic potentials (Munhoz and Frode, 2018; Unnikrishnan and Jayasri, 2018; Tang et al., 2020; Zhu et al., 2021), when these studies were conducted either at *in vitro* and/or *in vivo* experimental models. This particular topic of the journal has received numerous submissions but considering the focus of this special topic and the quality of the articles, only few of these were ultimately published including three comprehensive reviews, one mini review and three original articles. Out of many complications of diabetes mellitus, diabetic retinopathy is one of the leading causes of diabetes-associated blindness in the industrial world (Bhatwadekar et al., 2021). Chang et al. thoroughly discussed the roles of several non-coding RNAs such as microRNAs, lncRNAs and cricRNAs, involved in the gene expression as well as other biological processes in the development of novel therapeutics for diabetic retinopathy, which may help in developing novel therapeutics for diabetic retinopathy. The involvement of microRNAs has been further observed in one of the original studies published under this topic, where Guan et al. reported the upregulation of miR-199a-5p in the placenta of patients with gestational diabetes mellitus. On the other hand, while approximately 50% of people with diabetes are undiagnosed, diagnosing the disease at an early or a prediabetic stage can make a big difference in preventing or delaying the development of T2D. Applications of newly identified biomarkers may make a considerable difference in the diagnosis of prediabetes and ultimately preventing the rapidly growing prevalence of T2D. This has been widely discussed in the mini review published on this topic by Luis et al. As mentioned above, novel antidiabetic compounds are being identified either from the natural or synthetic origin, when a number of factors may limit the propagation of plants with potential antidiabetic phytochemicals for the viable production of antidiabetic drugs. Ali et al. not only elaborated on the recent progress of antidiabetic phytoconstituents but also their cellular mechanisms, and clinical outcomes as well as micropropagation methods of plants using callus culture and nano-elicitation strategies, which may help in the viable production of antidiabetic medicines from phytochemicals. Lastly, many medicines for different diseases have been developed *via* modulating the activities of cell membrane channels and gene expressions. It has been shown by Lu et al. in one of the original studies on this topic that Cisapride, a stomach-intestinal motility drug, may play a crucial role in reducing diabetes-associated hyperglycemia and hyperinsulinemia by modulating the expression of KCNH6, a potassium channel protein. This has not only been confirmed by *in vitro* experiments but also by using KCNH6 knockout mice. There is also another original study where the roles of D-Chiro-Inositol on hepatic insulin resistance has been investigated by Cheng et al., when Li et al. comprehensively reviewed the roles of pyroptosis, particularly mediated by the NLPR3, in the progression of T2D. In a world, all these reviews and original studies will contribute significantly to create better strategies for the development of novel anti-diabetic medicines by using molecular targets in the following years.
